# An Interference Mitigation Scheme of Device-to-Device Communications for Sensor Networks Underlying LTE-A

**DOI:** 10.3390/s17051088

**Published:** 2017-05-10

**Authors:** Jeehyeong Kim, Nzabanita Abdoul Karim, Sunghyun Cho

**Affiliations:** Department of Computer Science and Engineering, Hanyang University, 55 Hanyangdaehak-ro, Sangnok-gu, Ansan, Gyeonggi-do 426-791, Korea; manje111@hanyang.ac.kr (J.K.); anzabanita@gmail.com (N.A.K.)

**Keywords:** wireless sensor network (WSN), device-to-device (D2D), fractional frequency reuse (FFR), almost blank sub-frame (ABS), long-term evolution (LTE), signal to interference plus noise ratio (SINR)

## Abstract

Device-to-Device (D2D) communication technology has become a key factor in wireless sensor networks to form autonomous communication links among sensor nodes. Many research results for D2D have been presented to resolve different technical issues of D2D. Nevertheless, the previous works have not resolved the shortage of data rate and limited coverage of wireless sensor networks. Due to bandwidth shortages and limited communication coverage, 3rd Generation Partnership Project (3GPP) has introduced a new Device-to-Device (D2D) communication technique underlying cellular networks, which can improve spectral efficiencies by enabling the direct communication of devices in proximity without passing through enhanced-NodeB (eNB). However, to enable D2D communication in a cellular network presents a challenge with regard to radio resource management since D2D links reuse the uplink radio resources of cellular users and it can cause interference to the receiving channels of D2D user equipment (DUE). In this paper, a hybrid mechanism is proposed that uses Fractional Frequency Reuse (FFR) and Almost Blank Sub-frame (ABS) schemes to handle inter-cell interference caused by cellular user equipments (CUEs) to D2D receivers (DUE-Rxs), reusing the same resources at the cell edge area. In our case, DUE-Rxs are considered as victim nodes and CUEs as aggressor nodes, since our primary target is to minimize inter-cell interference in order to increase the signal to interference and noise ratio (SINR) of the target DUE-Rx at the cell edge area. The numerical results show that the interference level of the target D2D receiver (DUE-Rx) decreases significantly compared to the conventional FFR at the cell edge. In addition, the system throughput of the proposed scheme can be increased up to 60% compared to the conventional FFR.

## 1. Introduction

Wireless sensor networks (WSNs) have been used in various fields including environment monitoring, home automation, healthcare, agriculture, military, smart grids, and smart cars [1,2]. In these applications, sensors are equipped with wireless radio interfaces in order to form a wireless network and communicate with other sensors or a data aggregator. WSN will also play an important role to open the early Internet of Things (IoT) market [1,3].

Some technical barriers, which should be overcome to use WSN as a network infrastructure of the early IoT market, still remain open issues. A representative open issue is the autonomous communications among sensors. It is inefficient for a central node to control all sensors in WSN. Thus device-to-device (D2D) communication is considered as a rising technology in WSNs to solve this problem [4,5]. In D2D communications, sensor nodes or devices are able to communicate with each other through autonomous manner. D2D communication enables sensor nodes in close proximity to establish a direct link with each other as opposed to being routed by a controller or central node. Recently, D2D communication technologies have been actively studied in oneM2M standard for IoT [6,7] and 3rd Generation Partnership Project (3GPP) standard for Long Term Evolution Advanced (LTE-A) systems [8,9]. Another open issue of WSN to boost IoT is the network coverage extension and interworking with heterogeneous networks. The cellular networks can be an excellent candidate to overcome this problem. The cellular networks usually cover the majority of countries and interoperate with other networks. Thus, there is a growing trend to interwork WSNs with cellular networks.

For these backgrounds, this paper considers D2D communication technologies for wireless sensor networks. In particular, D2D communication technologies underlying LTE-A systems are investigated to apply wireless sensor networks to IoT [8,10]. D2D communication underlying LTE-A systems can be defined as a technology enabling direct communication between proximal sensor nodes or user equipment (UE). In D2D communication, sensor nodes do not need to pass through cellular infrastructural nodes such as an enhanced node-B (eNB) or mobility management entity (MME) to setup a D2D communication link. In the beginning of D2D study, integrating D2D in an LTE-A network was strongly supported by Qualcomm, which previously developed FlashLinQ. FlashLinQ is a proprietary technology which allows cellular devices to discover each other automatically and communicate with thousands of other FlashLinQ enabled devices within 1 km range [4,11]. To avoid the loss of network throughput in D2D communication underlying LTE-A systems, the licensed spectrum of cellular networks should be shared with D2D communications. Unfortunately, the interference among cellular user equipment (CUE) and D2D user equipment (DUE) is inevitable. Because of this, a considerable amount of research on the interference management has been conducted with regard to D2D communications being laid into LTE-A cellular networks where CUEs and DUEs share the same resources. In [12], the authors proposed a radio resource allocation scheme for D2D communication underlying cellular networks using strict fractional frequency reuse (FFR) to reduce the inter-cell interference of a DUE on receiving mode (DUE-Rx) at the cell edge area. In [13], the authors proposed the modified FFR to improve the coverage of CUEs and DUEs in cell edge areas. In this scheme, different resources were allocated to DUEs based on their location. Wenbin et al. proposed a resource allocation scheme in which dedicated frequency resource blocks (FRBs) are assigned to D2D links to avoid interference [14]. In [15], an intelligent power control mechanism has been proposed. It is based on the soft FFR that allocates radio resources to CUEs and DUEs with variable ratios. The drawback of prior systems, which are based on strict FFR, is the inefficient use of radio resources and the decrease of throughput. In the strict FFR, the entire frequency band can not be fully used in an entire cell. Some portion of resources are used only for CUEs or cell center users and the remaining portion is allocated to DUEs or cell edge users. Thus, the waste of resources and the loss of throughput are inevitable in the strict FFR. On the other hand, the entire frequency can be used for the entire users in the soft FFR, but the interference occurs from CUEs to DUE-Rx. The interference can prevent a DUE-Rx from receiving data from a CUE on transmitting mode (CUE-Tx) in cell edge areas.

In this paper, a hybrid mechanism of fractional frequency reuse and almost blank sub-frame (FFR-ABS) schemes are proposed to mitigate inter-cell interference caused by CUEs of neighboring cells to D2D pairs in cell edge areas. The proposed scheme can guarantee the Quality-of-Service (QoS) of both CUE and D2D pairs in terms of throughput and signal to interference and noise ratio (SINR), and increase the throughput of UEs in the cell edge area. The rest of this paper is organized as follows: Section 2 describes proximity services and resource allocation for D2D in LTE-A standards. The system model and proposed scheme are discussed in Section 3. Mathematical modeling, analysis, and discussion of the performance evaluations are provided in Section 4 and Section 5, respectively. Finally, concluding remarks are summarized in Section 6.

## 2. D2D Communications in LTE-A Standards

### 2.1. Proximity Service (ProSe) in LTE-A

Proximity service (ProSe) is a proximity based D2D service defined in 3GPP LTE-A Release 12 [16]. In ProSe, ProSe system architecture, discovery procedure, and D2D data services are defined. Unlike the existing network in which UEs connect to eNBs for data exchanging, UEs in ProSe can establish a direct D2D communication link without intervention of LTE core entities. In proximity services, UEs can select a D2D or cellular communication mode according to the channel quality. All UEs receive pilot signals broadcasted by eNBs on the downlink control channel. Then, UEs estimate the channel quality from eNBs in order to choose a communication mode between LTE and D2D. If the channel quality between UE and eNB is worse than it is between UEs, UE selects the D2D communication mode; otherwise, the cellular communication mode is used.

### 2.2. LTE-A Architecture for D2D

**Figure 1:**
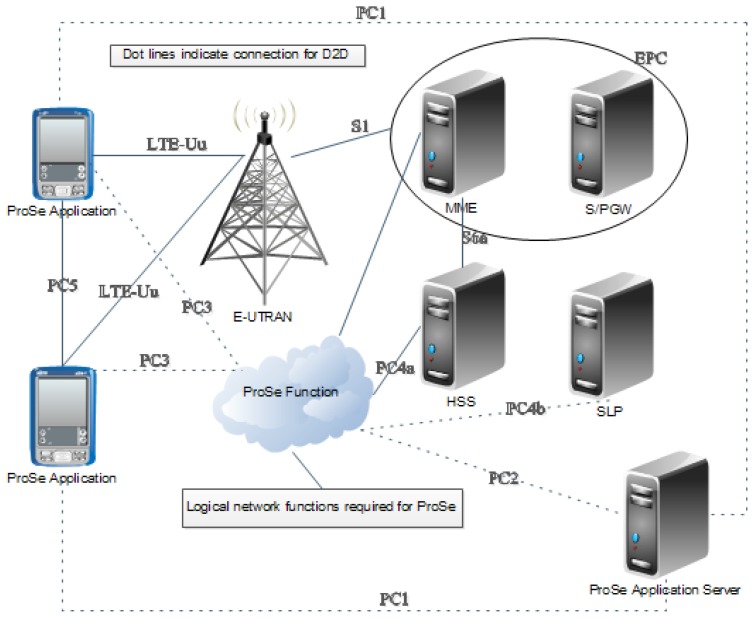
Long Term Evolution Advanced (LTE-A) Device-to-Device (D2D) Architecture.

In the D2D underlying LTE-A network system, it is important to maintain the conventional architecture of the LTE-A core system. For this reason, the two main parts of the LTE-A architecture, evolved terrestrial radio access network (E-UTRAN) and evolved packet core (EPC) are firstly described as shown in Figure 1. E-UTRAN is a radio interface defined as a part of LTE physical layer specification. The prime entity of E-UTRAN is evolved node-B (eNB) in the LTE system. EPC is a core network architecture that has the features of an all-IP network to provide high data rate and low latency. For D2D communications, there are five additional interfaces, referred to as PC1, PC2, PC3, PC4a, and PC4b, to connect with other LTE core entities [17]. A proximity-based service (ProSe) application server has been newly introduced for proximity services. It interoperates with a mobility management entity (MME), a home subscriber server (HSS), and a secure user plane location platform (SLP) to support ProSe functions as shown in Figure 1.

### 2.3. Proximity Discovery Scenarios and Interference

**Figure 2:**
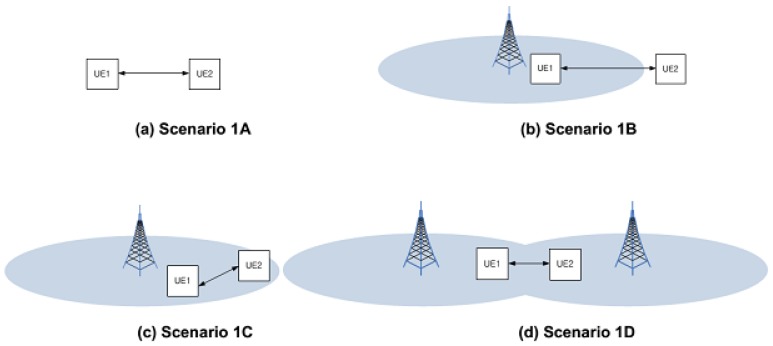
Proximity discovery scenarios.

A feasibility study with regard to proximity-based services (FS-ProSe, TR 22.803) defines proximity-service for UEs. The identified areas included services related to commerce and public safety that would be of interest to both operators and users. In Release 12 of the 3GPP standards [16], D2D discovery is designed to support different services that are bounded by discovery range. For this reason, D2D communication is also limited according to the D2D discovery boundary. Figure 2 shows the scenario for D2D ProSe where UE1 and UE2 are located in-coverage/out-coverage of a cell [16]. According to D2D connection scenarios, DUEs can be affected from the different interference sources. Table 1 presents network coverage combinations and interference sources for each proximity discovery scenarios depicted in Figure 2. Thus, to mitigate the interferences of D2D link efficiently, an interference mitigation scheme should consider all of the abovementioned scenarios and interference sources.

### 2.4. Resource Allocation for D2D Communications

Resource allocation (RA) is an important technical element that determines the interference level of DUEs and CUEs. The easiest way to avoid interference between D2D and cellular transmission modes is to allocate an unlicensed spectrum to DUEs. However, a licensed spectrum is allocated to D2D communications in LTE-A ProSe because assigning an unlicensed spectrum to D2D mode is impractical due to various technical problems. FFR is a typical method used to allocate the same radio resources to DUEs and CUEs. As described in the previous section, FFR is divided into strict FFR and soft FFR. Strict FFR can certainly avoid interference between DUE and CUE, but radio resource reuse is low. On the other hand, the entire radio resources can be dynamically reused to DUEs and CUEs, and, accordingly, the overall cell throughput increases in soft FFR. The drawback of soft FFR is that DUEs or CUEs can be suffered from inter-cell interference. Therefore, it is necessary to study an efficient resource allocation scheme for D2D communications that can efficiently mitigate interference without throughput loss.

## 3. System Model and Proposed Scheme

### 3.1. System Model

**Figure 3:**
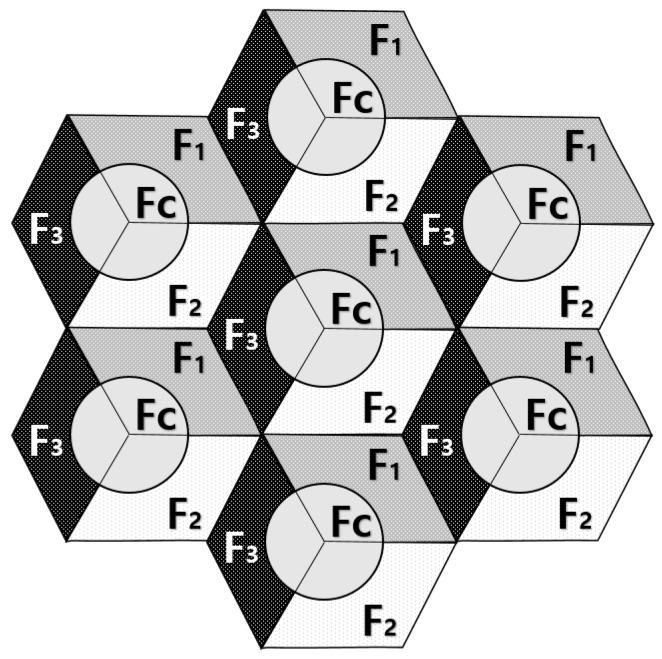
System model and resource allocation for soft fractional frequency reuse (FFR).

We considered a 7-cell hybrid network consisting of cellular user equipment (CUE) and D2D user equipment (DUE) with a spectrum-sharing D2D-enabled cellular network and focused on uplink transmission with regard to cellular users. The entire cell was divided into two main regions: a center cell region (inner region) and cell-edge region (outer region), as can be seen in Figure 3. All UEs were uniformly distributed within the cell where resources and power for cellular links and D2D links were controlled by the eNB. The network structure used soft fractional frequency reuse (FFR). The entire frequency spectrum was used in a single cell and the same frequency spectrum was reused in the neighboring cells. For soft FFR, each cell was divided into a cell-center (inner area) region and cell-edge (outer area) region. The entire frequency band was divided into four portions, namely, $S_{0}$, $S_{1}$, $S_{2}$, and $S_{3}$, with the corresponding bandwidth represented as $F_{c}$, $F_{1}$, $F_{2}$, and $F_{3}$, as can be seen in Figure 3. To make sure that each CUE in the same cell or in neighboring cells did not interfere with each other, resources were allocated in an orthogonal manner. Through the use of a soft FFR scheme, interference among CUEs was mitigated efficiently within the network.

### 3.2. The Proposed Interference Mitigation Scheme

We propose a hybrid scheme of FFR and almost blank sub-frame (ABS) to mitigate the inter-cell and inter-sector interferences. The proposed scheme firstly adopts soft FFR for efficient frequency reuse and interference avoidance between cellular and D2D links. The proposed scheme also exploits the ABS method to mitigate the inter-sector interference that can not be efficiently managed by only soft FFR.

The overall operational flow of the proposed scheme is described in Algorithm 1. The proposed scheme consists of three phases: soft FFR, ABS, and data transmission. In phase 1, an eNB operates soft FFR. The eNB divides its own cell into four different sectors like $S_{0}$, $S_{1}$, $S_{2}$, and $S_{3}$. Then, it checks the locations of CUEs and allocates frequency to CUEs through the soft FFR method. The details of soft FFR are described in the next section. Phase 2 is an ABS configuration by the eNB. If the eNB receives a D2D connection request from DUE-Tx *i* to DUE-Rx *j*, it lists CUEs whose distance to the DUE-Rx *j* is less than $D_{max}^{ABS}$. The list is a set ${\mathrm{ABS}}_{j}^{CUE}$ of CUEs that should be silent on ABS sub-frames for the DUE-pair i,j. The eNB then sends control signals including the ABS pattern for the DUE-pair i,j. The final phase is data transmission by DUEs and CUEs. In this phase, the eNB checks the DUE-pair i,j and the set ${\mathrm{ABS}}_{j}^{CUE}$ for the DUE-pair i,j. The CUEs in the set should be silent during ABS sub-frames. In the proposed scheme, the CUEs that cause severe interference are selectively chosen to be silent in ABS to minimize the throughput loss.
**Algorithm 1** Operations of the Proposed Scheme.1:**Parameters:** $D_{max}^{ABS}$2:**Input:** DUE-Tx *i*, DUE-Rx *j*   **Phase1 – Soft FFR by eNB**3:sectorize its cell into $S_{0}$, $S_{1}$, $S_{2}$, and $S_{3}$4:check locations of CUEs5:allocate frequency for CUE with soft FFR   **Phase2 – ABS by eNB**6:**while** receive D2D connection request from DUE-Tx *i* to DUE-Rx *j* **do**7:    ${\mathrm{ABS}}_{j}^{CUE}$ = { CUE *c*| distance between CUE *c* and DUE-Rx *j* < $D_{max}^{ABS}$ }8:    send control signal to DUE-Tx *i* including ABS pattern for the DUE-pair i,j9:**end while**   **Phase3 – Data transmission by DUEs and CUEs**10:**for** DUE-pair i,j that requested D2D connections **do**11:    **if** ${\mathrm{ABS}}_{j}^{CUE}\neq \emptyset$ **then**12:        **for** CUE *c* in ${\mathrm{ABS}}_{j}^{\mathrm{CUE}}$ **do**13:            mute data transmission of CUE *c* on ABS subframes14:        **end for**15:    **end if**16:**end for**

**Figure 4:**
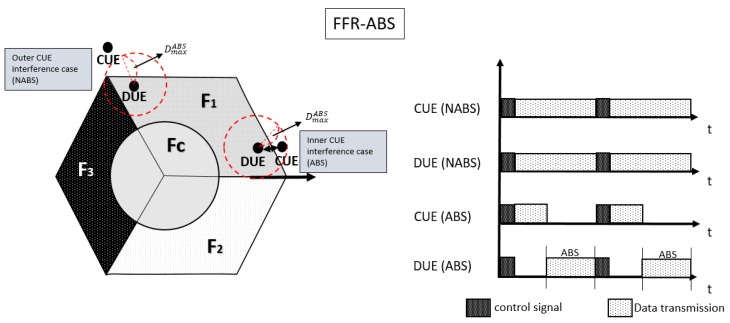
Hybrid FFR-almost blank sub-frame (ABS) system.

Figure 4 depicts different interference sources and the sub-frame allocation for DUE-pair and CUE in the proposed FFR-ABS scheme. In the proposed FFR-ABS, ABS is applied only to CUEs whose distance is too close to D2D links. As shown in Figure 4, there are two different interference sources such as outer and inner CUEs. If the distance to the D2D link is longer than $D_{max}^{ABS}$, the node is called an outer CUE. Since the signal from an outer CUE does not interfere with the D2D link, the ABS method is not applied to an outer CUE. It is defined as a non-ABS (NABS) case in the proposed scheme. In an NABS case, even outer CUEs transmit data on the non-orthogonal frequency with DUEs, and the interference to DUEs can be tolerable. On the other hand, a CUE that is closer to the D2D link than $D_{max}^{ABS}$ is called an inner CUE. In the proposed scheme, ABS is applied to an inner CUE because it can severely interfere with the D2D link. An eNB broadcasts ABS patterns to the entire cell. In the proposed FFR-ABS scheme, DUE-Tx transmits data on the ABS subframes while inner CUEs should be silent on the ABS subframes to avoid interference with D2D links as shown in Figure 4.

#### 3.2.1. Soft Fractional Frequency Reuse

Interference management is a major challenge with regard to deploying D2D communication into LTE-A cellular networks. In the proposed scheme, soft fractional frequency reuse (FFR) is used as an interference management method between CUEs and DUEs as shown in Table 2. A serving eNB can manage the interference between CUE and DUE pairs using soft FFR. According to Table 2, D2D pairs are allowed to use any partition of frequency ($F_{c}$, $F_{1}$, $F_{2}$, and $F_{3}$) except that which is allocated to its CUE in the same area. In this way, D2D pairs can reuse the same frequency with CUEs located in the neighbor sectors and avoid interference from CUEs in the same sector. However, D2D pairs located at the cell edge area can experience serious interference from CUEs of neighbor sectors because those are allowed to use any frequency except the one allocated to CUEs in the same sector. To resolve the inter-sector interference caused by CUEs of neighbor sectors, the proposed scheme exploits the ABS method with soft FFR.

#### 3.2.2. Almost Blank Sub-Frame

**Figure 5:**
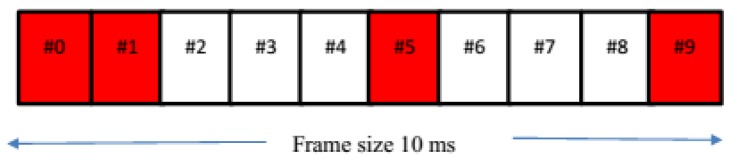
Frame structure with ABS.

The proposed scheme mitigates the inter-sector interference of a target DUE-Rx using the ABS method. In the proposed scheme, a DUE on receiving mode (DUE-Rx), which receives severe interference, is regarded as a victim node. On the other hand, a CUE in a neighboring sector, which causes the inter-sector interference, is treated as an aggressor. To address the inter-sector interference problem in the LTE-A network, 3GPP Release 10 proposed the enhanced Inter-Cell Interference Coordination (eICIC) technique by the allocation of almost blank sub-frames (ABSs) [18]. ABS is a technique based on adaptive resource partitioning in the time domain as can be seen in Figure 5. In Figure 5, sub-frames 2, 3, 4, 6, 7, and 8 are ABS where the aggressor nodes do not send data signals to avoid interference to victim nodes. During ABS, the aggressors can transmit only control signals such as cell-specific reference signals (CRSs) [19,20]. In Figure 5, sub-frames 0, 1, 5, and 9 are not blank, which are reserved to transmit primary synchronization signals (PSS), secondary synchronization signals (SSS), SIB-1, and paging.

We propose a hybrid FFR-ABS scheme to mitigate the inter-sector interferences as well as inter-cell interferences. CUEs in cell-edge areas should have much higher transmission power to communicate with eNB located in the cell center. Thus, CUEs also cause interferences to DUE-Rxs in neighboring sectors. The proposed FFR-ABS scheme can prevent this problem and improve overall throughput.

## 4. Mathematical Modeling and Analysis

### 4.1. Stochastic Geometry Model for Wireless Sensor Networks

We use a stochastic geometry model as a numerical tool to analyze interference, connectivity, and coverage in large-scale wireless sensor networks [21]. Recently, stochastic geometries have also been employed with regard to modeling D2D-enabled cellular networks [22,23,24]. We consider Poisson point processes (PPP) to model the D2D sensor networks’ underlying cellular systems. We also model SINR distributions where interference management schemes are not considered. Table 3 describes the notations used in the proposed mathematical models.

To derive the distribution of network elements, we assume that eNBs are distributed with homogeneous PPP $\Phi_{b}$ of intensity $\lambda_{b}$. CUEs are located by independent stationary point process $\Phi_{c}$ with density $\lambda_{c}$. It is assumed that UE-Txs are distributed in a homogeneous PPP $\Phi_{d}$ with density $\lambda_{d}$. DUE-Rxs, the receiver of D2D communication, are uniformly distributed following DUE-Tx during the interval [$D_{min}$, $D_{max}$]. ${\mathrm{ABS}}_{j}^{CUE}$ is a set of CUEs whose distance to DUE-Rx *j* is less than $D_{max}^{ABS}$. The CUEs in ${\mathrm{ABS}}_{j}^{CUE}$ should be silent mode on ABS subframes. Similarly, ${\mathrm{ABS}}_{c}^{DUE}$ is also defined as a set of DUE-Tx *i* in case the distance between CUE *c* and the DUE-Rx *j* is less than $D_{max}^{ABS}$. The reference distance is the distance between CUE *c* and DUE-Rx *j*. Therefore, the set of DUE-Tx *i* that interferes with the CUE *c* is determined by the distance between DUE-Rx *j* and CUE *c*.

### 4.2. Channel and Link Distance Models

The channel and link distance models are defined by signal power. The received signal power of CUE, $P_{j,c}^{CUE}$, from CUE *j* to eNB *c*, can be derived as follows:

(1) $$P_{j,c}^{CUE}=G_{j,c}^{CUE}p^{CUE},$$

where $G_{c,j}^{CUE}={\left|H_{c,j}^{CUE}\right|}^{2}$. Similarly, the received signal power of DUE-Rx from DUE-Tx *i* to DUE-Rx *j*, $P_{i,j}^{D2D}$, can be defined as follows:

(2) $$P_{i,j}^{D2D}=G_{i,j}^{D2D}p^{D2D},$$

where $G_{i,j}^{D2D}={\left|H_{i,j}^{D2D}\right|}^{2}$.

### 4.3. Signal-to-Interference-Plus-Noise-Ratio (SINR) Model

This section defines the SINR of a target DUE-Rx that suffers from interferences caused by CUEs. Regarding the D2D communication scheme, the interference to a target DUE-Rx comes from co-channel DUEs and other DUE-Txs in the same cell or neighboring cells. Therefore, the SINR of DUE-Rx *j* from DUE-Tx *i* with the conventional FFR can be expressed as follows:

(3) $$SINR_{FFR}^{D2Dn}=\frac{P_{{}_{i,j}}^{D2D}}{\gamma^{2}+\sum_{k\in K_{tx}^{n},k\neq i}P_{k,j}^{D2D}+\sum_{k\in C^{n}}P_{k,j}^{CUE}}{|}_{i,j\in Fn}.$$

Similarly, the SINR of CUE with the conventional FFR can be defined as:

(4) $$SINR_{FFR}^{CUEn}=\frac{P_{{}_{i,c}}^{CUE}}{\gamma^{2}+\sum_{k\in K_{tx}^{n}}P_{k,c}^{D2D}+\sum_{k\in C^{n}}P_{k,c}^{CUE}}{|}_{i\in Fn}.$$

In the proposed FFR-ABS mode, DUE-Rx does not suffer from CUEs whose distance to DUE-Rx is less than $D_{max}^{ABS}$ because those do not transmit data simultaneously through the ABS method. Thus, the SINR of DUE-Rx *j* from DUE-Tx *i* in the FFR-ABS can be defined as follows:

(5) $$SINR_{ABS}^{D2Dn}=\frac{P_{{}_{i,j}}^{D2D}}{\gamma^{2}+\sum_{k\in K_{tx}^{n},k\neq i}P_{k,j}^{D2D}+\sum_{k\in C^{n},k\notin {\mathrm{ABS}}_{j}^{CUE}}P_{k,j}^{CUE}}{|}_{i,j\in Fn}.$$

Note that CUE *k* does not interfere with DUE-Rx *j* in ${\mathrm{ABS}}_{j}^{CUE}$. In the same way, the SINR of CUE in the FFR-ABS can be described as follows:

(6) $$SINR_{ABS}^{CUEn}=\frac{P_{{}_{i,c}}^{CUE}}{\gamma^{2}+\sum_{k\in K_{tx}^{n},k\notin {\mathrm{ABS}}_{c}^{DUE}}P_{k,c}^{D2D}+\sum_{k\in C^{n}}P_{k,c}^{CUE}}{|}_{i\in Fn}.$$

### 4.4. Optimal ABS Ratio

In order to maximize the throughput of CUEs and DUEs, the ABS ratio should be optimally determined. Based on mathematical analysis of Shannon formula, we define data rate *R* as follows:

(7) $$R_{mode}^{link}=log_{2}\left(1+SINR_{mode}^{link}\right),$$

where a link can be a D2D or cellular link. To be short, a cellular link can be denoted as CUE and be applied to FFR or FFR-ABS. FFR-ABS is shortened as ABS. From Equation (7), we define the total network throughput to maximize the data rate of CUE and DUE. Firstly, the total network throughput of conventional FFR can be expressed as the following:

$$\begin{array}{ccc} R_{total}^{FFR} & = & \sum R_{FFR}^{D2D}+\sum R_{FFR}^{CUE}. \end{array}$$

In FFR-ABS, a part of CUEs have ABS applied and others operate by the FFR method. The nodes that are on the ABS scheme have to transmit data on their subframes only. Thus, the total data rate of FFR-ABS is as follows:

(8) $$\begin{array}{ccc} R_{total}^{ABS} & = & \beta (Pr_{ABS}^{D2D}\sum R_{ABS}^{D2D}+\left(1-Pr_{ABS}^{D2D}\right)\sum R_{FFR}^{D2D})\\ & & +\left(1-\beta \right)(Pr_{ABS}^{CUE}\sum R_{ABS}^{CUE}+\left(1-Pr_{ABS}^{CUE}\right)\sum R_{FFR}^{CUE}), \end{array}$$

where $Pr_{ABS}^{D2D}$ = $\frac{n({⋃}_{c\in C}{\mathrm{ABS}}_{c}^{D2D})}{n(K)}$, and $Pr_{ABS}^{CUE}$ = $\frac{n({⋃}_{j\in K}{\mathrm{ABS}}_{j}^{CUE})}{n(C)}$. These are the ratios of DUE and CUE that have the ABS scheme applied. β is an ABS ratio. It means how many subframes are used as ABS in a frame. We can calculate the instantaneous data rate with Shannon capacity equation, but the instantaneous data rate can not consider time resource distribution for a node. In order to apply the distribution of time resources to the data rate, we use an ABS ratio, β. During the ABS period, an eNB exchanges an ABS ratio via X2 interface. There are two prime interfaces in LTE such as X2 and S1 interfaces. The X2 interface is used to communicate between eNBs. The eNBs share the information for UEs, hand-over, channel status, and the configuration of eNBs [25].

## 5. Performance Evaluations

### 5.1. Simulation and Path Loss Models

In order to evaluate the performance of the proposed FFR-ABS, we use both mathematical analysis and computer simulation implemented by *Python*. In the simulator, a 2-tier cell structure that consists of seven hexagonal cells is assumed. An eNB at the center cell acts as a resource controller for both CUEs and DUEs. The number of CUEs per cell is four and they are uniformly distributed. There are also pairs of DUEs in each cell. Each D2D pair consists of a D2D transmitter (DUE-Tx) and its corresponding D2D receiver (DUE-Rx). Thus, four pairs of DUEs and eight DUEs are uniformly distributed in each cell. DUE-Tx and the target DUE-Rx are separated by a uniform random variable *x* that varies between 20–50 m in distance within each cell. Computational parameters and their values are given in Table 4. The main criterion for an eNB to allow a piece of user equipment to operate in either cellular mode or D2D mode is the location of a sender and a receiver vis-à-vis the eNB. Thus, path loss measurement facilitates determining the connection mode of UEs. Path-loss is modeled according to micro-urban models in the International Telecommunication Union Radio-communication Sector (ITU-R) reports [26]. By applying different path-loss models to DUEs and eNBs relaying CUEs as [27], the path-loss of the micro-urban models for DUEs ($PL_{D2D}$) and eNBs relaying CUEs ($PL_{eNB}$) cab be expressed as follows:

$$\begin{array}{ccc} \left(\mathrm{NLOS}\;100\%\right)\;PL_{D2D} & = & 24.82+35.31\times log_{10}d,\\ \left(\mathrm{NLOS}\;80\%\;+\;\mathrm{LOS}\;20\%\right)\;PL_{D2D} & = & 28+40\times log_{10}d,\\ \left(\mathrm{NLOS}\;100\%\right)\;PL_{eNB} & = & 31.25+33.76\times log_{10}d,\\ \left(\mathrm{NLOS}\;80\%\;+\;\mathrm{LOS}\;20\%\right)\;PL_{eNB} & = & 30.35+36.7\times log_{10}d, \end{array}$$

where *d* is the distance between transmitter and receiver in meters. Channel models usually consider an urban model with non-line-of-sight (NLOS). However, we additionally consider NLOS 80% + line of sight (LOS) 20% channel models to analyze the performance of the proposed scheme.

### 5.2. Simulation Results

To evaluate the performance of the proposed scheme, we consider three scenarios:

1. Scenario 1: Different channel models
2. Scenario 2: Different transmission powers
3. Scenario 3: Different traffic loads

**Figure 6:**
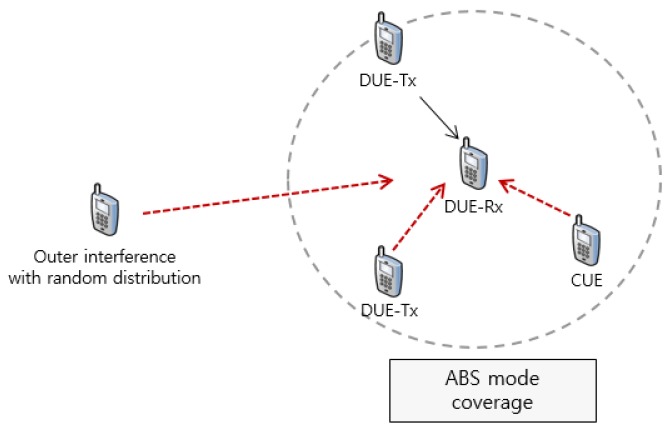
Scenarios 1 and 2: Isolated D2D user equipment (DUE)-pairs and cellular user equipment (CUE) model.

**Figure 7:**
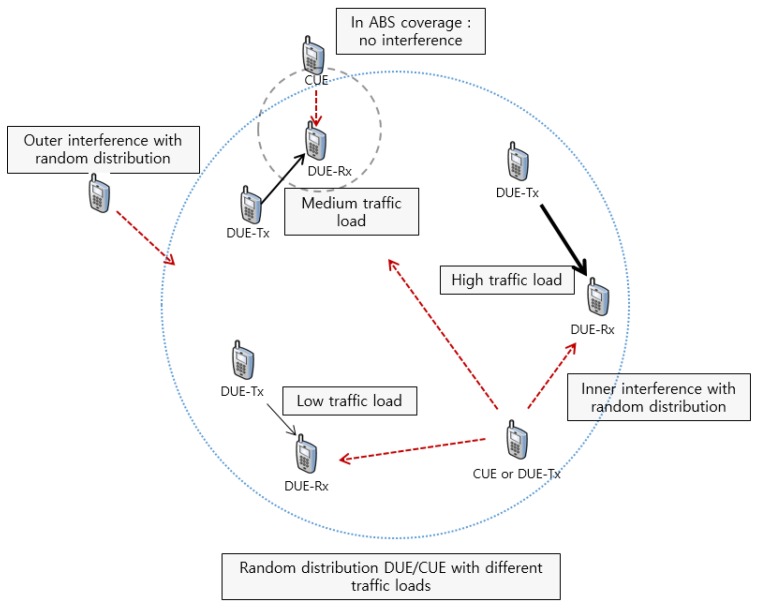
Scenario 3: Random distribution with typical traffic loads.

Figure 6 and Figure 7 depict these scenarios. In Figure 6, there are two significant interferers to ABS mode coverage like DUE-Tx and CUE. In this case, the interference from CUE can be suppressed through the FFR-ABS scheme, but interference from DUE-Tx cannot be avoided. We simulate this model with various transmission powers and channel models. Figure 7 describes a model in which all of the DUEs and CUEs are randomly distributed. In this model, we compare throughput for each node with different traffic loads.

#### 5.2.1. Scenario 1: Different Channel Model

In this section, we evaluate the performance of the proposed scheme through a numerical computational analysis. We firstly perform the evaluation of the isolated D2D pair at the cell edge area when a D2D pair shares resources with a CUE located in a neighboring cell. In addition, we compare the performance of the proposed scheme (FFR-ABS) with the conventional FFR proposed in [15]. In this scenario, we assume that a target DUE-Rx is located 20 m from its corresponding DUE-Tx, and its power is set to 20 dBm (100 mW). The locations of interferers (DUE and CUE) are randomly designated between 20–60 m. During the mathematical analysis, we compare the results obtained for the conventional system using normal sub-frames (FFR only) and the proposed FFR-ABS to protect the victims from CUE interference.

**Figure 8:**
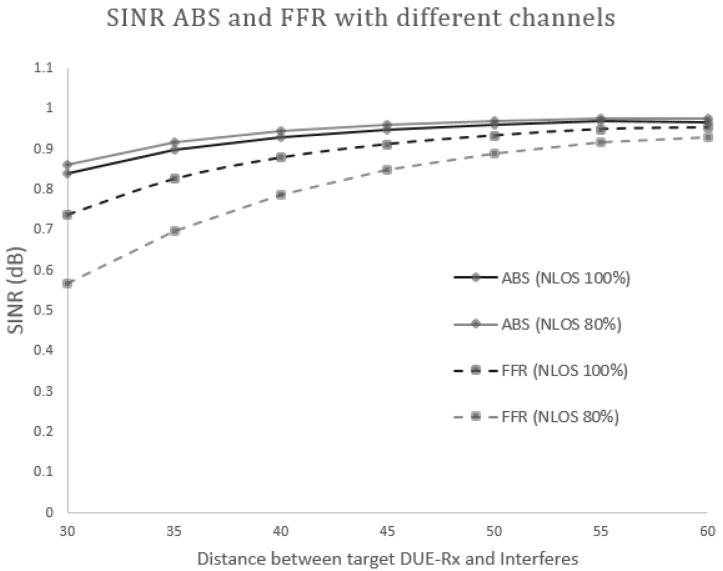
Combined graphs of signal to interference and noise ratio (SINR)-FFR and SINR-ABS received at the target DUE-Rx.

**Figure 9:**
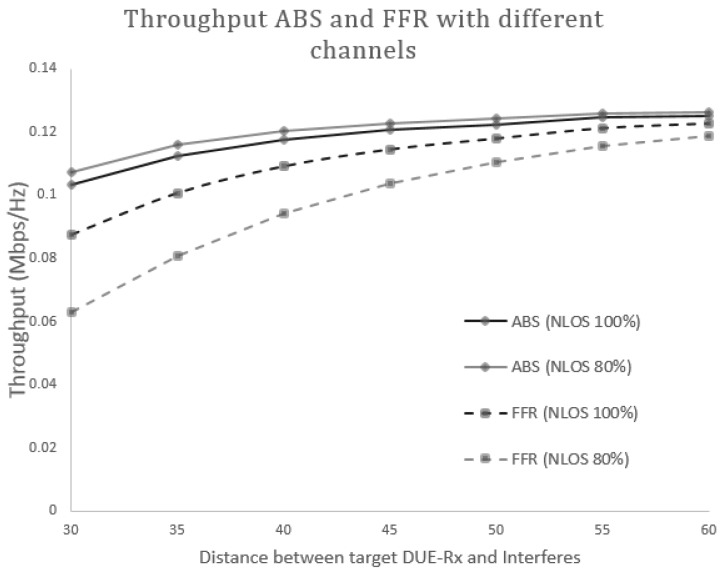
Combined graphs of FFR and ABS throughput received at the target DUE-Rx.

Figure 8 and Figure 9 show the SINR and the received throughput at the target DUE receiver (DUE-Rx). The SINR and the throughput of the proposed scheme is higher compared to the conventional method because a certain spatial distance can be maintained between DUE-Rx and the interferers (CUEs and DUE pairs). In the conventional system, the target DUE-Rx experiences strong interference from the CUE in neighboring cells that share the same resources. The proposed scheme also achieves a higher SINR at the cell edge area as opposed to the existing scheme. This is due to usage of the hybrid FFR-ABS mechanism; during ABS time, the CUE would not transmit data. The muting sub-frames would help the target DUE-Rx to receive dedicated data from its corresponding DUE-Tx. As shown in Figure 8 and Figure 9, as the interferers are located closer to the target DUE-Rx, less SINR and throughput are gained at the target DUE-Rx. Therefore, the proposed scheme achieves better performance than the conventional scheme in terms of the received SINR at the target DUE-Rx. As seen in Figure 9, the throughput can be increased up to 60% by the proposed FFR-ABS in case the distance between the target DUE-Rx and interferer is less than 30 m.

#### 5.2.2. Scenario 2: Different Transmission Power Levels for CUE and DUE

**Figure 10:**
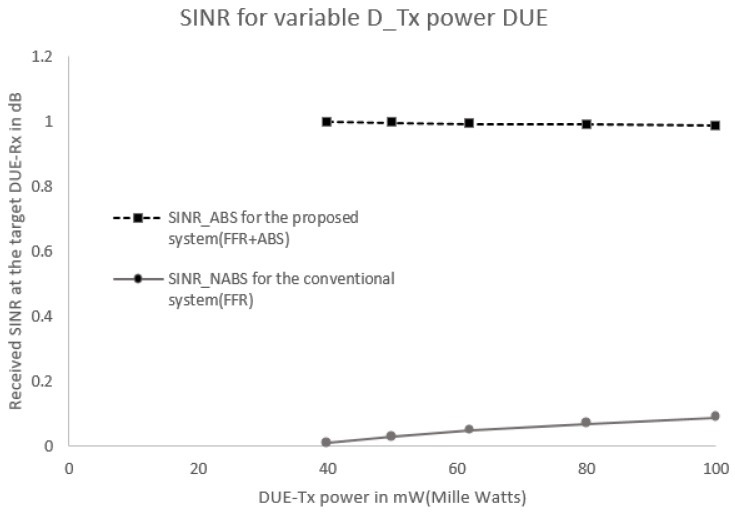
SINR received at the target DUE-Rx with constant CUE-Tx and variable DUE-Tx power levels.

**Figure 11:**
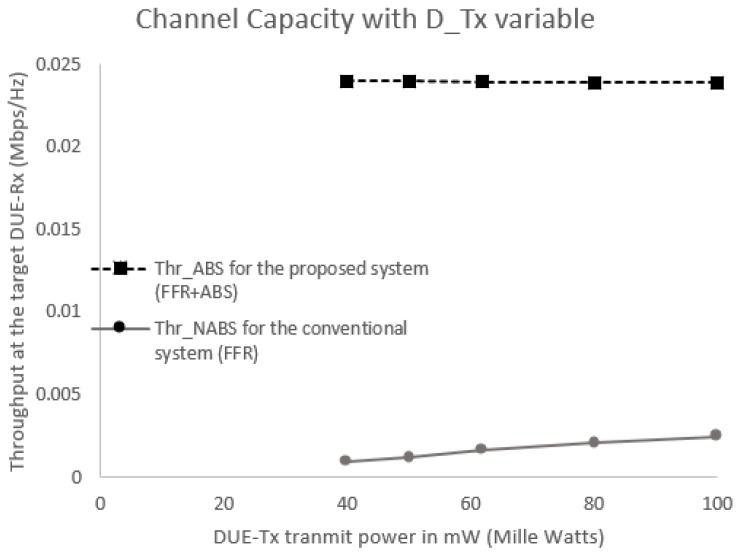
Channel capacity received at the target DUE-Rx with constant CUE-Tx and variable DUE-Tx power levels.

Figure 10 and Figure 11 show the SINR and throughput at DUE-Rx when aggregate interference from all interfering nodes are present in the network. In this scenario, all DUEs use the same transmission power levels (100 mW or 20 dBm), even those that are located at different distances; the CUEs also exhibited the same transmission power levels (250 mW or 24 dBm). Figure 10 and Figure 11 show that the received SINR and throughput of the target DUE-Rx are higher in the proposed FFR-ABS compared to those of the conventional FFR at the cell edge area.

**Figure 12:**
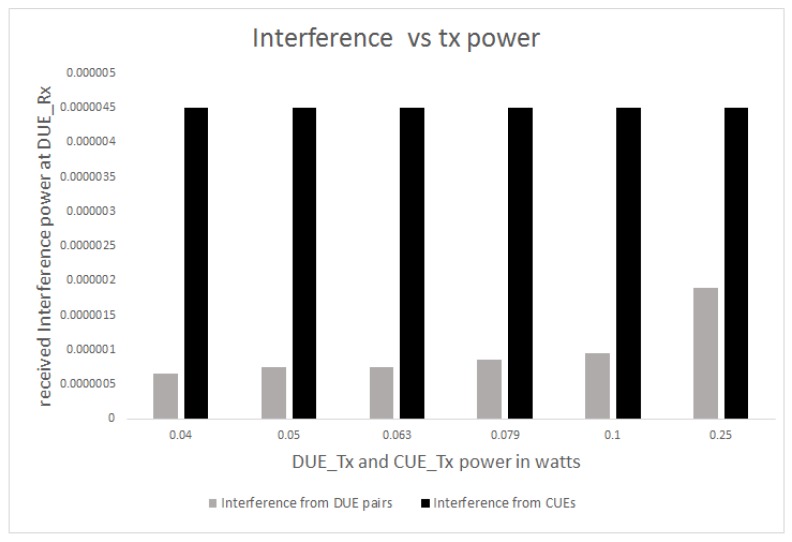
Interference levels from both interferers for variable Tx power.

Figure 12 shows the throughput received at the target DUE-Rx with respect to the variable DUE transmission power. It is obvious from Figure 12 that the proposed scheme achieves higher throughput for the target DUE-Rx in the cell edge area compared to the conventional scheme. As shown in Figure 12, CUEs experience much higher interference levels compared to DUE pairs. Even at the same transmission power levels, CUEs outperform the DUE pairs. Thus, the use of the proposed hybrid FFR-ABS can reduce aggregate interference that could reach the target DUE-Rx. Once aggregate interference to the target DUE-Rx is significantly reduced, SINR and throughput can be significantly increased for any DUE transmission power level, as shown in Figure 12.

**Figure 13:**
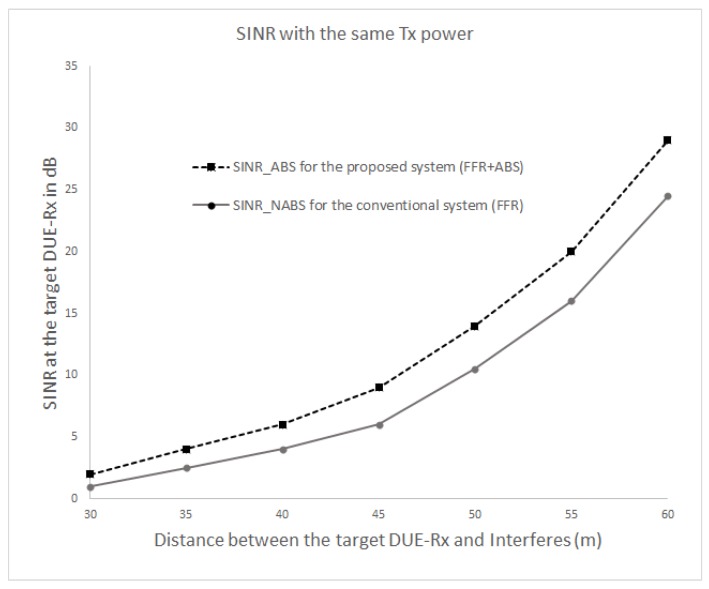
SINR received at the target DUE-Rx under the same CUE-Tx and DUE-Tx power levels.

**Figure 14:**
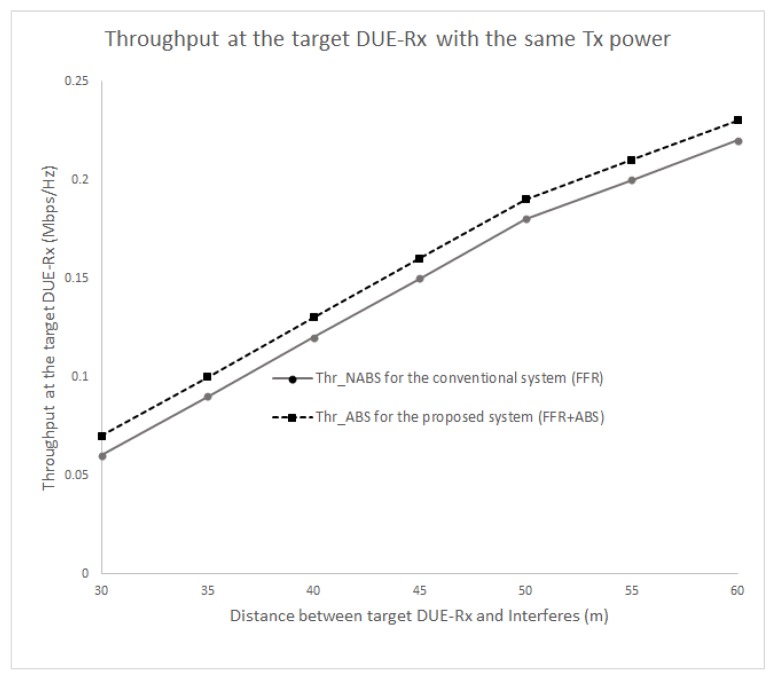
Channel capacity received at the target DUE-Rx under the same CUE-Tx and DUE-Tx power levels.

We also assume equal transmission power levels for the target transmitter (DUE-Tx) and all interferers (DUEs and CUEs). As shown in Figure 12, when the same transmission power levels are applied, aggregate interference increases due to a high level of interference from the CUEs. Therefore, for the same transmission power levels, the interference of target DUE-Rx would increase depending on the location of the interferers. The closer the interferer, the higher the aggregate interference, resulting in a reduction to the SINR and throughput. By applying the ABS, interference from the CUEs can be suppressed during ABS time. Due to non-CUE transmission, aggregate interference is reduced, but SINR and throughput are increased as shown in Figure 13 and Figure 14.

#### 5.2.3. Scenario 3: Different Traffic Loads for CUE and DUE

**Figure 15:**
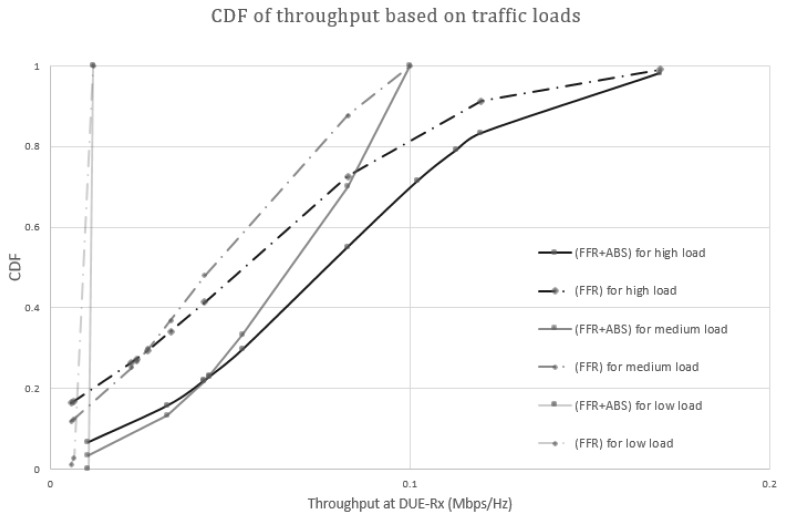
Cumulative distribution function (CDF) of throughput based on traffic loads.

In Scenario 3, we consider high, medium, and low traffic loads in CUEs and DUEs. Real-time video streaming services can be a high traffic load service. Web browsing and voice call can be typical examples of medium traffic load and low traffic load services, respectively. According to [28], we can simplify the traffic loads to 242 kbps, 100 kbps, and 12 kbps, respectively. Figure 15 shows CDF of throughputs based on the traffic loads. The proposed and conventional schemes achieve almost the same throughput under a low traffic load. However, the proposed scheme shows higher throughput compared to the conventional FFR under medium and high traffic loads. In the FFR-ABS scheme, interferences from nearby CUEs can be ignored because the CUEs are muted in ABS. Thus, DUE-Rxs have better throughput in the proposed FFR-ABS than in the conventional FFR.

## 6. Conclusions

In this work, we proposed a hybrid mechanism with regard to FFR and ABS for D2D communication underlying the LTE-A network. The hybrid mechanism provided a solution toward mitigating serious interference caused by CUEs of neighboring cells to D2D receivers in the cell edge area. The use of ABS in the conventional FFR provided an effective method with regard to decreasing inter-cell interference levels to victims (DUE-Rx) near the cell edge area. Based on our mathematical analysis, we determined that the ABS ratio that could provide balance between CUEs and DUEs in terms of Quality-of-Service. Performance evaluation results of the proposed scheme showed that the introduction of the FFR-ABS scheme could significantly improve system throughput and guarantee Quality-of-Service to both CUE and D2D pairs in the cell edge area. Further studies should be extended from the scheme to minimize intra-cell interference caused by co-channel D2D pairs.

## Figures and Tables

**Table 1:** Device-to-Device (D2D) scenarios and interference sources.

	UE1	UE2	Serving Cell	Communication Path	Interference Sources
**1A**	Out	Out	n/a	direct	n/a
**1B**	In	Out	n/a	direct	Cellular User Equipments (CUEs) in the same cell
**1C**	In	In	Same cell	direct	Cellular User Equipments (CUEs) in the same celland D2D User Equipments (DUEs) in the same celland neighboring cells
**1D**	In	In	Different cell	direct	Cellular User Equipments (CUEs) in the same celland D2D User Equipments (DUEs) in the same celland neighboring cells

**Table 2:** Resource allocation for cellular user equipment (CUE) and device-to-device (D2D) pairs.

Area	Partition	Frequency	CUE	D2D
Cell-Center	$S_{0}$	$F_{c}$	$F_{c}$	$F_{1}$, $F_{2}$ and $F_{3}$
Cell-Edge	$S_{1}$	$F_{1}$	$F_{1}$	$F_{c}$, $F_{2}$ and $F_{3}$
Cell-Edge	$S_{2}$	$F_{2}$	$F_{2}$	$F_{c}$, $F_{1}$ and $F_{3}$
Cell-Edge	$S_{3}$	$F_{3}$	$F_{3}$	$F_{c}$, $F_{1}$ and $F_{2}$

**Table 3:** Notations.

Notation	Definition
$\Phi_{b}$	Homogeneous Poisson point processes (PPP) for eNB distribution
$\Phi_{c}$	Homogeneous Poisson point processes (PPP) for CUE distribution
$\Phi_{d}$	Homogeneous Poisson point processes (PPP) for DUE-Tx distribution
$\lambda_{b}$	Intensity of homogeneous Poisson point processes (PPP) for eNB distribution $\Phi_{b}$
$\lambda_{c}$	Intensity of homogeneous Poisson point processes (PPP) for CUE distribution $\Phi_{c}$
$\lambda_{d}$	Intensity of homogeneous Poisson point processes (PPP) for DUE-Tx distribution $\Phi_{d}$
$H_{x,y}^{CUE}$,$H_{x,y}^{D2D}$	Complex channel gain between node x to y for cellular and D2D links, respectively
$K_{tx}^{n}$,$K_{rx}^{n}$	A set {1,...,K} of K DUE-Tx/DUE-Rx terminals using D2D links for frequency $F_{n}$
$C^{n}$	A set {1,...,C} of C UE using cellular links for frequency $F_{n}$
$D_{min}$, $D_{max}$	The minimum/maximum distance between DUE-Tx and DUE-Rx, respectively
$D_{max}^{ABS}$	Maximum distance between CUE and DUE-Rx for adopting ABS scheme
${\mathrm{ABS}}_{j}^{CUE}$	A set of CUEs whose distance from DUE-Rx *j* is less than $D_{max}^{ABS}$
${\mathrm{ABS}}_{c}^{DUE}$	A set of DUEs whose distance from CUE *j* is less than $D_{max}^{ABS}$
$\gamma^{2}$	Additive White Gaussian Noise (AWGN) noise variance
$p^{CUE}$, $p^{D2D}$	Transmission power for CUE and DUE-Tx, respectively
β	ABS pattern ratio

**Table 4:** Computational parameters.

Parameter	Assumption/Value
Cellular layout	Hexagonal grid, 7 cells sites
Path Loss Model (D2D-NLOS 100%)	$24.82+35.31\times log_{10}d$
Path Loss Model (D2D-NLOS 80%)	$28+40\times log_{10}d$
Path Loss Model (CUE-NLOS 100%)	$31.25+33.76\times log_{10}d$
Path Loss Model (CUE-NLOS 80%)	$30.35+36.7\times log_{10}d$
CUE transmit power	24 dBm
DUE transmit power	20 dBm
Noise power density	−174 dBm
Inter-site distance (radius of the cell)	500 m
Carrier frequency	2 GHz
Bandwidth	10 MHz
Number CUEs	10
Number of DUE	20
ABS pattern period	10 ms
Distance between D2D	20–60 m
Traffic patterns	Full-buffered
Monte Carlo number	10,000

## References

[1] Ericsson (2011). More Than 50 Billion Connected Devices.

[2] Cho S., Choi J.W., You C. (2013). Adaptive multi-node multiple input and multiple output (MIMO) transmission for mobile wireless multimedia sensor networks. Sensors.

[3] Evans D. (2011). The Internet of Things How the Next Evolution of the Internet is Changing Everything.

[4] Mumtaz S., Rodriguez J. (2014). Smart Device to Smart Device Communication.

[5] LG Electronics (2014). Discussion on D2D Discovery Physical Layer Design.

[6] Swetina J., Lu G., Jacobs P., Ennesser F., Song J. (2014). Toward a standardized common M2M service layer platform: Introduction to oneM2M. IEEE Wirel. Commun..

[7] OneM2M Alliance. http://www.onem2m.org/.

[8] Feng J. (2013). D2D Communication in LTE-Adavanced Network. Ph.D. Dissertation.

[9] The Mobile Broadband Standard. http://www.3gpp.org/DynaReport/FeatureOrStudyItemFile-580038.htm.

[10] Lin M., Ouyang J., Zhu W.P. (2016). Joint Beamforming and Power Control for Device-to-Device Communications Underlaying Cellular Networks. IEEE J. Sel. Areas Commun..

[11] Ma C., Liu J., Tian X., Yu H., Cui Y., Wang X. (2015). Interference Exploitation in D2D-Enabled Cellular Networks-A Secrecy Perspective. IEEE Trans. Commun..

[12] Chae H.S., Gu J., Choi B.G., Chung M.Y. Radio Resource Allocation Scheme for Device-to-Device Communication in Cellular Networks Using Fractional Frequency Reuse. Proceedings of the 17th Asia-Pacific Conference on Communications (APCC).

[13] Zhang Z., Hu R.Q., Qian Y., Papathanassiou A., Wu G. D2D communication underlay uplink cellular network with fractional frequency reuse. Proceedings of the 11th International Conference on the Design of Reliable Communication Networks (DRCN).

[14] Wu W., Zhang Y. Dedicated resource allocation for D2D communications in cellular systems employing FFR. Proceedings of the 6th International Conference on Wireless Communications and Signal Processing (WCSP).

[15] Shah S.T., Gu J., Hasan S.F., Chung M.Y. (2015). SC-FDMA-based resource allocation and power control scheme for D2D communication using LTE-A uplink resource. EURASIP J. Wirel. Commun. Netw..

[16] 3GPP TR 23.703. http://www.arib.or.jp/english/html/overview/doc/STD-T63v11_00/5_Appendix/Rel12/23/23703-c00.pdf.

[17] 3GPP TS 23.303 v12.2.0 Release 12. http://www.etsi.org/deliver/etsi_ts/123300_123399/123303/12.02.00_60/ts_123303v120200p.pdf.

[18] Chen Y.F., Ding W.S., Wang L.C. Hybrid Protected Subframes Resource Allocation and Throughput Estimation in LTE-A HetNet. Proceedings of the IEEE International Conference on Internet of Things, Green Computing and Communications (GreenCom), and Cyber, Physical and Social Computing (CPSCom).

[19] Kshatriya S.N.S., Kaimalettu S., Yerrapareddy S.R., Milleth K., Akhtar N. On interference management based on subframe blanking in heterogeneous LTE networks. Proceedings of the 5th International Conference on Communication Systems and Networks (COMSNETS).

[20] Jiang L., Lei M. Resource allocation for eICIC scheme in heterogeneous networks. Proceedings of the IEEE 23rd International Symposium on Personal, Indoor and Mobile Radio Communications (PIMRC).

[21] Ma C., Wu W., Cui Y., Wang X. On the performance of successive interference cancellation in D2D-enabled cellular networks. Proceedings of the IEEE Conference on Computer Communications (INFOCOM).

[22] Lin X., Andrews J.G. Optimal spectrum partition and mode selection in device-to-device overlaid cellular networks. Proceedings of the IEEE Global Communications Conference (GLOBECOM).

[23] Lin X., Ratasuk R., Ghosh A., Andrews J.G. (2014). Modeling, analysis, and optimization of multicast device-to-device transmissions. IEEE Trans. Wirel. Commun..

[24] Ye Q., Al-Shalash M., Caramanis C., Andrews J.G. Device-to-device modeling and analysis with a modified Matern hardcore BS location model. Proceedings of the IEEE Global Communications Conference (GLOBECOM).

[25] Ghosh R., Ratasuk R. (2011). Essentials of LTE and LTE-A.

[26] Guidelines for Evaluation of Radio Interface Technologies for IMT-Advanced. http://www.itu.int/pub/R-REP-M.2135.

[27] Xing H., Hakola S. The investigation of power control schemes for a device-to-device communication integrated into OFDMA cellular system. Proceedings of 21st Annual IEEE International Symposium on Personal, Indoor and Mobile Radio Communications (PIMRC).

[28] Raheem R., Lasebase A., Loo J. Performance Evaluation of LTE networking via using Fixed/Mobile Femtocells. Proceedings of the 28th International Conference on Advanced Information Networking and Applications Workshops.

